# Lithium and Atypical Antipsychotics: The Possible WNT/β Pathway Target in Glaucoma

**DOI:** 10.3390/biomedicines9050473

**Published:** 2021-04-26

**Authors:** Alexandre Vallée, Jean-Noël Vallée, Yves Lecarpentier

**Affiliations:** 1 Department of Clinical Research and Innovation (DRCI), Foch Hospital, 92150 Suresnes, France; 2 Centre Hospitalier Universitaire (CHU) Amiens Picardie, Université Picardie Jules Verne (UPJV), 80054 Amiens, France; valleejn@gmail.com; 3 Laboratoire de Mathématiques et Applications (LMA), UMR CNRS 7348, Université de Poitiers, 86000 Poitiers, France; 4 Centre de Recherche Clinique, Grand Hôpital de l’Est Francilien (GHEF), 6-8 rue Saint-Fiacre, 77100 Meaux, France; yves.c.lecarpentier@gmail.com

**Keywords:** WNT/β-catenin pathway, lithium, atypical antipsychotics, inflammation, oxidative stress, glutamatergic pathway, glaucoma

## Abstract

Glaucoma is a progressive neurodegenerative disease that represents the major cause of irreversible blindness. Recent findings have shown which oxidative stress, inflammation, and glutamatergic pathway have main roles in the causes of glaucoma. Lithium is the major commonly used drug for the therapy of chronic mental illness. Lithium therapeutic mechanisms remain complex, including several pathways and gene expression, such as neurotransmitter and receptors, circadian modulation, ion transport, and signal transduction processes. Recent studies have shown that the benefits of lithium extend beyond just the therapy of mood. Neuroprotection against excitotoxicity or brain damages are other actions of lithium. Moreover, recent findings have investigated the role of lithium in glaucoma. The combination of lithium and atypical antipsychotics (AAPs) has been the main common choice for the treatment of bipolar disorder. Due to the possible side effects gradually introduced in therapy. Currently, no studies have focused on the possible actions of AAPs in glaucoma. Recent studies have shown a down regulation of the WNT/β-catenin pathway in glaucoma, associated with the overactivation of the GSK-3β signaling. The WNT/β-catenin pathway is mainly associated with oxidative stress, inflammation and glutamatergic pathway. Lithium is correlated with upregulation the WNT/β-catenin pathway and downregulation of the GSK-3β activity. Thus, this review focuses on the possible actions of lithium and AAPs, as possible therapeutic strategies, on glaucoma and some of the presumed mechanisms by which these drugs provide their possible benefit properties through the WNT/β-catenin pathway.

## 1. Introduction

Glaucoma is a progressive neurodegenerative disease and one of the major cause of irreversible blindness. The number of worldwide glaucoma patients will increase from 76.5 million in 2020 to 111.8 million by 2040, mainly due to aging population [1,2]. Glaucoma presents the loss of retinal ganglion cells (RGCs), thinning of the retinal nerve fiber layer, and cupping of the optic disc [3]. Glaucoma is formed by heterogeneous diseases showing varying clinical presentations. Aging, high intraocular pressure (IOP), and a genetic causes are the major risk factors for glaucoma [3]. Primary open-angle glaucoma (POAG) is the major presentation in countries. However, 30% of patients have normal tension glaucoma (NTG) [4]. The etiology of POAG is well-known with mechanical and/or vascular mechanisms. The mechanical process implicates compression of the axons due to increased IOP, while the vascular mechanism shows events in which blood flow and ocular perfusion pressure are decreased to the posterior pole leading to damage [5,6]. Vascular or perfusion dysregulations in NTG show different clinical features, including migraine headaches, Raynaud’s phenomenon, or sleep apnea [7]. In high IOP glaucoma, both the anterior and posterior segments are affected, as extensive affection is detectable in the trabecular meshwork (TM) and along the inner retina-central visual pathway [8]. 

Lithium, introduced in 1949, is the most used drug for chronic mental illness, including bipolar disorder with depressive and manic cycles. Lithium remains a first-line treatment for bipolar disorder, manic-depressive illness, [9], traumatic brain injury [10], and numerous neurodegenerative diseases, such as Alzheimer’s, Huntington’s, and Parkinson’s diseases [11]. In acute treatment of mania, the efficacy of lithium is well established [12]. Numerous studies have presented that lithium can diminish manic relapses, even if its efficacy was lower in reduced depressive relapses [13]. In parallel, some studies have shown that lithium may diminish suicides and suicide attempts in patients suffering from mood disorders [14]. Lithium therapeutic mechanisms remain complex, including several pathways and gene expression, such as neurotransmitter and receptors, circadian modulation, ion transport, and signal transduction processes [15]. Recent studies show that the benefits of lithium extend beyond just the therapy of mood. Neuroprotection against excitotoxicity or brain damage are other action of lithium [16]. Moreover, recent findings have investigated the role of lithium in glaucoma [17,18] but its actions remain unclear. Nevertheless, recent studies have highlight possible mechanisms of lithium action through the WNT/beta-catenin pathway in glaucoma [19,20]. The combination of lithium and atypical antipsychotics (AAPs) has been the main common choice for the treatment of bipolar disorder [21]. Due to the possible side effects of the first-generation antipsychotics, the second-generation antipsychotics (also called AAPs) were gradually introduced in therapy [22]. Currently, no studies have focused on the possible actions of AAPs in glaucoma. 

Thus, this review focuses on the possible actions of lithium and AAPs as possible therapeutic strategies for glaucoma and some of the presumed mechanisms by which these drugs provide their possible benefit properties through the WNT/β-catenin pathway. 

## 2. Pathophysiology of Glaucoma

In primary open-angle glaucoma (POAG), responsible for IOP elevation, the IOP upregulation implicates the TM occlusion inducing by the iris tissue [8]. The chronic contact between the TM and iris leads to permanent affection of the TM. TM dysregulation and its diminished cellularity are the first stage to high tension glaucoma (HTG). Numerous factors, including oxidative stress (OS) and aging, as well as environmental factors, are implicated as the promotors of TM damage [23]. OS could be enhanced in the morphological alterations of the TM of glaucomatous eyes, due to it stimulating inflammatory response. Chronic inflammation and OS modulate each other, creating a vicious circle influencing the cellular responses. The cultures of TM presented a NF-ϰB pathway activation after exogenous stimulation such as IL-1 or H_2_O_2_. The NF-ϰB pathway stimulation leads to a significant expression of the endothelial leukocyte adhesion molecule-1 (ELAM-1), IL-1β and IL-6 [24]. ELAM-1 belongs to selectin families, which are cell adhesion molecules. ELAM-1 presence in POAG is a main factor for the onset of TM endothelial dysregulation [25]. In glaucoma, a progressive loss of TM cells was observed, due to the combination of both aging and stress conditions [26]. In HTG, the TM dysregulation involves both inflammation and reprogramming mechanisms with OS damage and endothelial dysregulation [27]. IL-6, IL-1, and TNF-alpha induce ECM reprogramming and alter cytoskeletal interactions in the glaucomatous TM [25]. 

Elevation of the IOP, at the lamina cribrosa or the optic nerve head (ONH) step, involves hypoperfusion and damages in reperfusion [28]. Increase in IOP is considered as a major factor of retinal ganglion cells (RGCs) dysfunction, leading to a retrograde transport blockade and the accumulation of neurotrophic factors at the lamina cribrosa instead of reaching the RGC soma [29]. The etiology of POAG remains unclear but numerous risk factors have been shown as causes of its onset, including increased IOP, aging, gender, family history, OS, systemic and ocular vascular factors, and inflammation [30]. The dysfunctions in the protein patterns shown in the aqueous humor (AH) of POAG patients is the consequence of the progressive loss of TM integrity [31]. TM-derived proteins can damage both the retina and optic nerve head (ONG) behavior in the posterior segment of the eye, leading to apoptotic signaling for RGCs and their axons in the ONH. The TM is the most sensitive tissue of the anterior segment of the eye to oxidative stress [32]. Glaucomatous TM cells presented POAG-typical molecular modifications, including ECM accumulation, cell death, dysfunction of the cytoskeleton, advanced senescence, NF-ϰB pathay activation and inflammatory markers release [24,33]. These results could suggest that the IOP elevation is associated to OS and degenerative processes affecting the human TM endothelial cells (hTMEs). The chronic exposure of TM cells to OS leads to numerous changes in the lysosomal pathway responsible for autophagia [34], as well as cell senescence with an increase in senescence-associated-galactosidase [35]. OS leads to lysosomal dysfunctions and the defective proteolytic activation of lysosomal enzymes with a subsequent diminution in autophagic flux and the activation of cell senescence [8]. 

## 3. Oxidative Stress, Inflammation and Glutamate in Glaucoma

Pathogenic processes of the neurodegenerative mechanism lead to the mechanical and vascular stress enhancing mitochondrial dysregulation, chronic oxidative stress (OS) and metabolic stress [36,37], excitotoxicity [38], and neuro-inflammation [39,40]. OS and cell senescence are increased in the aging retina [41,42] and are considered as the main glaucoma risk factors. In the aging retina, OS leads to the stimulation of a para-inflammation [43]. Para-inflammation, in glaucoma, is characterized by a tissue adaptive response to noxious stress [43]. However, a physiological stage of para-inflammation is needed to maintain homeostasis but when tissue is exposed to a chronic stress, inflammation may have a negative role and could be involved in both initiation and progression of the disease [44]. The deregulation of para-inflammation in the retina is a response to stress stimuli especially chronic OS. However, excessive and uncontrolled para-inflammation could implicated inflammatory responses with a release of cytokines/chemokines leading to neuroretina damages [45]. Para-inflammatory dysregulation could be associated to TM dysfunction and increased resistance to aqueous outflow, the main cause of increased IOP in POAG [8]. 

The mechanisms of reactive oxygen species (ROS) production are activated in several pathological conditions of the retina, such as glaucoma, occlusion of the central artery of the retina and the age-macular degeneration. They are enzymes, including the nicotinamide adenine dinucleotide phosphate (NADPH) oxidase, the cytochrome P450, the mitochondrial cytochrome oxidase, the xanthine oxidoreductase, and the eNOS decoupled, catalyzing the stimulation of ROS production in the vascular system tissues [46,47]. OS diminishes BH4 bioavailability, but increases BH2, which possessing cofactor activity to compete with BH4 for enhancing eNOS [48].

The TM was the main pathological region of PAOG [49]. IOP can be control by the balance between the production and out flow of the aqueous humor. The TM is composed by layers of trabecular beams and surrounded by elastic fibers, fibronectin and laminin. Abnormalities of the ECM are involved in high IOP [50]. Recently, the WNT/β-catenin pathway have been found to be associated with the development of glaucoma in the TM [51]. 

To date, the visual loss processes are not entirely elucidated in glaucoma, the ROS production plays an important role in its development [52]. ROS production rates are increased in patients with glaucoma in the acute mood but also in the blood serum [53]. In retinal arteries, a moderately increased IOP leads to ROS production, activation of NOX2 expression, and endothelial dysfunction, leading to the idea of IOP stimulation can damage the vascular function of the retina [54]. Nevertheless, some pathogenic mechanisms are linked to glaucoma, including glutamate excitotoxicity [55], which are not necessarily associated with the elevated levels of IOP [52]. It seems that the death of RGCs during a glaucoma lesion stimulates ROS production in vitro [56]. It has been shown that the ROS production controls the immune response by stimulating the action of antigen glial cells [56]. ROS production affects the retina, and increase the IOP to induce a dysfunction of the support glia, which facilitates the secondary degeneration of the RGCs in glaucoma [57]. The glial cells produced by ROS that affect the retina, and the PIO elevated to induce a dysfunction of the support glide, which facilitates the secondary degeneration of the CGR in the glaucoma [57]. 

The immune system is controlled by numerous inflammatory factors, including tumor necrosis factor α (TNF-α), interleukin-6 (IL-6), vascular endothelial growth factor (VEGF) and tumor growth factor-β (TGF-β) [58]. Inflammation leads to the stimulation of cyclooxygenase 2 (COX-2) [59]. Several cytokines (TNF-α, IL-1) stimulate COX-2 [60]. COX-2 activates ROS production [59,61]. The nuclear factor-ϰB (NF-ϰB) pathway can activate numerous factors leading to COX-2 and inducible nitric oxide synthase (iNOS) over-expressions [62]. Numerous findings have presented that NF-ϰB pathway can activate the expression of both TNF-α, IL-6, IL-8, STAT3, COX-2, BCL-2 (B-cell lymphoma 2), metalloproteinases (MMPs), VEGF [62], and then the overstimulation of the ROS production [63]. Moreover, iNOS is stimulated during chronic inflammation [64].

Numerous studies have presented the mechanism by which OS can activate chronic inflammation [65]. The imbalance caused by OS involves damages of signaling in cells [66]. ROS have a main role both upstream and downstream of NF-κB and TNF-α pathways. The hydroxyl radical is the main harmful of all the ROS. A vicious circle has been observed between ROS and these pathways. ROS are controlled by the NOX system. Furthermore, the modified proteins by ROS may involve the enhancement of the auto-immune response to activate TNF-α and NOX [67]. Nuclear factor erythroid-2 related factor 2 (Nrf2) is mainly associated to OS and inflammation [65]. Nrf2 is a transcription factor binding to the antioxidant response element (ARE) [68]. Numerous studies have shown that Nrf2 could have an anti-inflammatory role through the regulation of MAPK, NF-ϰB, and PI3K pathways [69]. Then, Nrf2 could have a main action against OS damages [70]. Moreover, evidence also have shown that mitochondrial dysfunction could have a significant action in cancer processes [65].

Glutamate is an amino-acid responsible for the brain’s primary excitatory neurotransmission. Glutamate is considered as the main neurotransmitter within the cortico-striatal-thalamic circuit involved in OCD [71]. Glutamatergic neurons are embedded in every brain circuit in comparison to dopamine and serotonin which are used by a small minority of neural cells in the brain. Glutamate is the main excitatory neurotransmitter in brain and is present in more than 50% of synapses. This signaling plays a major role for neuronal plasticity, memory, and learning [72]. Rapid neurotoxicity enhanced by neuronal excitotoxin has been observed with abnormal glutamate levels [73]. In neurons, glutamate is stored in synaptic vesicles from which it is released. Glutamate release increases glutamate concentration in the synaptic cleft to bind ionotropic glutamate receptors. SLC1A1 encodes for the neuronal excitatory Na+-dependent amino acid transporter 3 (EAAT3). EAAT1 and EAAT2 are the main astrocyte glutamate transporters whereas EAAT3 is the major neuronal glutamate transporter. Glutamate is converted into glutamine in astrocytes and released. Then, glutamine is take up by neurons to be re-converted into glutamate [74]. The role of the EAAT3 is to control glutamate spillover which affects pre-synaptic N-methyl-D-asparate (NMDA) and metabotropic glutamate receptors activity [75,76]. EAAT3 activity is dysregulated by the overexpression of GSK-3β [77].

In glaucoma, the glutamate pathway dysregulation may enhance RGC death and has been shown to be controlled by the NMDA receptor that, due to its higher Ca2+ permeability, could have a great affinity for glutamate and a slower inactivation [78,79]. In retinal neurodegeneration, the glutamate excitotoxicity is involved in the mtDNA damage or DNA oxidation–related mitochondrial dysfunction [80]. Glutamate excitotoxicity activation in the excitatory signaling leading to neuronal cell death by high levels of glutamate and the over-stimulation of NMDA receptors. The excitotoxic damages to RGCs may be enhanced by the augmentation of glutamate synthesis or the diminution of glutamate clearance [81]. 

## 4. WNT/β-Catenin Pathway

The WNT name is derived from Wingless drosophila melanogaster and its mouse homolog Int. WNT/β-catenin pathway is implicated in several mechanisms and controlling signaling, including embryogenesis, cell proliferation, migration and polarity, apoptosis, and organogenesis [82]. Nevertheless, during several pathological diseases, the WNT/β-catenin pathway can be altered, including inflammation, metabolic, neurological and psychiatric disorders, fibrosis and cancer processes [83].

**Figure 1:**
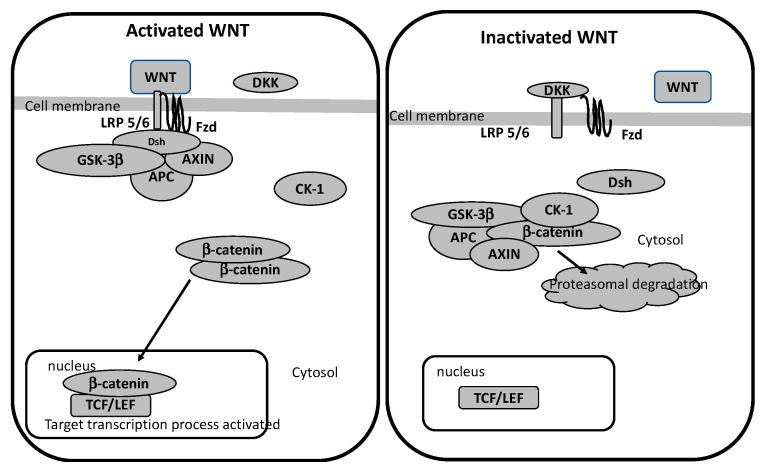
Activated and Inactivated WNT pathway. APC: adenomatous polyposis coli; CK-1: casein kinase 1; DKK: dickkopf-1; Dsh: disheveled; Fzd: frizzled; GSK-3β: glycogen synthase kinase-3β; LRP 5/6: low-density lipoprotein receptor-related protein 5/6; TCF/LEF: T-cell factor/lymphoid enhancer factor; TNF- α: tumor necrosis factor alpha.

The WNT pathway belongs to the family of secreted lipid-modified glycoproteins [84]. WNT ligands are produced by neurons and immune cells localized in the CNS [85]. WNT pathway dysfunction could affect numerous neurodegenerative pathologies [86,87,88,89,90]. The WNT pathway has a main stage called as the β-catenin/T-cell factor/lymphoid enhancer factor (TCF/LEF). The cytoplasmic accumulation of β-catenin is modulated by the destruction complex AXIN, tumor suppressor adenomatous polyposis coli (APC), and glycogen synthase kinase-3 (GSK-3β). With absence of WNT ligands, the destruction complex has a role in the hyper-phosphorylation of the cytoplasmic β-catenin and leads to its proteasomal destruction. Nevertheless, in their presence, the WNT ligands bind to Frizzled (FZL) and LDL receptor-related protein 5/6 (LRP 5/6) to interrupt the destruction complex and prevents β-catenin degradation into the proteasome. β-catenin translocates to the nucleus to interact with the TCF/LEF. This stimulates WNT target genes [91,92,93] (Figure 1). 

Glycogen synthase kinase-3β (GSK-3β) is one of the major inhibitors of the WNT/β-catenin pathway [94,95,96,97,98,99]. As an intracellular serine-threonine kinase, GSK-3β is a major negative controller of the WNT signaling [100]. GSK-3β is implicated in the control of numerous kinds of pathophysiological pathways, including cell membrane signaling, cell polarity, and inflammation [101,102,103]. GSK-3β interacts by downregulating the cytoplasmic β-catenin and stabilizing it to enhance its nuclear migration. Inflammation is an age-related mechanism correlated with the activation of GSK-3β pathway and the diminution of the WNT/β-catenin pathway [104]. 

Recent studies have observed that glaucoma patients presented an activation of the GSK-3β pathway and its downregulation may be an interesting therapy target [105,106]. Dysregulation of GSK-3β is implicated in the pathogenesis of numerous pathologies, such as neuropsychiatric disorders [107]. GSK-3β is a regulator of numerous signaling including inflammation, neuronal polarity, or either cell membrane signaling [102]. GSK3β is known to be the major inhibitor of the canonical WNT/β-catenin pathway [98,108,109,110,111,112]. 

### WNT/β-Catenin Pathway in Glaucoma

Recent findings have presented that the WNT/β-catenin pathway is involved in the pathophysiology of TM cells and that this pathway could serves as a regulator of IOP [113]. Secreted frizzled-related protein 1 (sFRP1), a WNT inhibitor, is stimulated in the glaucomatous TM, and the exogenous sFRP1 implicates high IOP [114,115]. In sFRP1-perfused eyes, the level of β-catenin was downregulated [51]. sFRP1 activity is correlated with cell stiffness [115]. TM cells possess several responses to the stimulus of different concentrations of sFRP1 [115]. It has been shown that sFRP1 is activated in normal TM cells grown on substrates activating the stiffness of the glaucomatous TM. The augmentation of stiffness of the TM implicates the aqueous humor out flow resistance and lead to IOP elevation [115]. Furthermore, the GSK3β, can diminish the activity of the WNT/β-catenin pathway and lead to ocular hypertension in association with sFRP1 [114]. It has been shown that there may be two effects of WNT in glaucoma [113]. The glaucoma gene myocilin (MYOC) has been shown to be a regulator of WNT/β-catenin pathway [116]. Nevertheless, the damaging effects of MYOC mutation on the WNT pathway remain unclear in the TM. The aqueous humor out flow resistance is affected by the change in adhesion junctions and cell contact [113]. The WNT/β-catenin pathway could be a novel target for the therapy of glaucoma [117]. Numerous WNT target genes are expressed in the TM, and the WNT ligand WNT3a is disrupted [113,114]. The over-activation of both sFRP1 or Dkk1 can lead to the augmentation of IOP in perfusion-cultured eyes [113,114]. Furthermore, the co-therapy with a small-molecule WNT pathway stimulator can diminish sFRP1-induced OHT in eyes. The stimulation of WNT/β-catenin pathway in the TM using lithium chloride decreases the production of some ECM and matricellular proteins [19,118]. WNT/β-catenin pathway and K-cadherin are main regulator of the IOP, and the decrease of these pathways can elevate the IOP in glaucoma [119]. Recent findings have presented that activation of the WNT/β-catenin pathway increases the fibrosis-associated proteins in the TM and that the POAG-associated WNT antagonist sFRP1 activates ECM deposition, TM cell stiffness [115] and IOP [113,114]. Furthermore, recent studies have presented that the WNT/β-catenin may control TM homeostasis and IOP by a cross-inhibit circle with TGF-β signaling [118]. 

## 5. Lithium and AAPs in Glaucoma

### 5.1. Lithium in Glaucoma

Very few studies have investigated the actions of lithium in glaucoma. Lithium can act through several intracellular signaling including GSK-3β [120,121]. Its therapeutic effects are observed after a long-term of administration. Lithium can protect cells against several pathways including glutamate and deprivation of serum and nerve growth factors [120]. Lithium acts on RGCs to enhance neuronal survival and axonal regeneration at the treatment concentrations (0.5–1.2 mM) [17]. Lithium may be used a treatment drug to act on retinal and optic nerve neurodegeneration, such as glaucoma and RGC loss [17,122]. Numerous findings have shown that high doses of lithium may lead to irreversible neurotoxicity damages [123]. Excessive intake or impaired excretion may be the consequence of lithium accumulation. Lithium is mainly susceptible to be accumulated in bone, muscle, liver, thyroid, and kidney [124]. Dehydration, febrile illness, or gastrointestinal loss may be involved by increased lithium levels in serum [125]. Renal toxicity is mainly common in people with chronic lithium therapy with nephrogenic diabetes insipidus [126]. Neurologic effects are hyperreflexia, nystagmus, or ataxia and remains mostly reversible [125]. Other troubles are reversible cardiovascular effects (QT prolongation, intraventricular conduction defects) [127], gastrointestinal effects [128], and endocrine effects [129]. But, low doses of lithium are correlated with lower side-effects [130].

Lithium induces Bcl-2 transcription in retinas. Bcl-2 is a main controller for the regulation of both neural survival and axonal regeneration [131]. Moreover, the mechanism of Bcl-2 control of apoptosis [132]. Lithium can stimulate the PI3K/Akt pathway to upregulate the expression of Bcl-2 [90]. Recent findings have shown that neuroprotection by lithium can occur through inhibition of the NMDA receptor and glutamate-induced AKT activity [133]. Lithium downregulates DRP1 through GSK3-β inhibition, to reduce mitochondrial fission [105]. Excessive mitochondrial fission can lead to the dysregulation of the electron transport chain and oxidative phosphorylation, leading to apoptosis [134]. Moreover, lithium promotes RGCs survival and axon regeneration [17,135]. Several mediators, including N-methyl-D-aspartate receptors, PI3K/Akt pathway, cytoprotective Bcl-2, and GSK-3β are implicated in the process underlying lithium-induced neuroprotection [136]. Nevertheless, the underlying mechanisms have not been fully elucidated and remain unknown. 

### 5.2. AAPs in Glaucoma

Second generation antipsychotic drugs (also called AAPs) are known for their cardiovascular side effects including hypotension by the alpha 1 adrenoceptor blockage [137]. The alpha 1 adrenoceptor, responsible for vasoconstriction, have been found to have several subtypes such as alpha 1A, 1B, and 1D [138]. Alpha 1A receptor can produce a positive inotropic effect leading to blood vessel constriction. Alpha 1A receptor is also implicated in central hypotensive responses [138]. The AAPs, including clozapine [139], quetiapine [140], and risperidone [141], were shown to lead to hypotension by inhibiting alpha 1 receptors. Iloperidone, a AAP drug, presents binding affinity to serotoninergic (5-HT2A, 5-HT6 and 5-HT7), dopaminergic (D2, D3 and D4) and adrenergic (α1 and α2C) receptors in the CNS) [142]. Iloperidone can inhibit serotonergic 5HT2A receptor (J-13) and adrenergic alpha 1A receptor [143,144] and is responsible for hypotension [145]. In dose 0.03 mg/Kg i.p., Iloperidone present reduced blood pressure within the 10 min of administration for animals [146]. Only drug-induced angle-closure glaucoma is of direct relevance for AAPs administration. 

As previously described, ROS production has a main role in glaucoma physiopathology. Risperidone, which possesses a canonical antipsychotic pharmacological process, can control the pro-inflammatory response [147,148] by decreasing OS in schizophrenic patients [149]. This antipsychotic can diminish the OS and rescue synaptic plasticity in PFC pyramidal cells from schizophrenia-like animal model [150]. Risperidone, can decrease iNOS expression and can stimulate SOD activity in brain areas [151]. This suggests that risperidone and other AAPs can decrease OS in glaucoma [148,152]. Among AAPs, clozapine and olanzapine can also decrease OS [148,153]. Nevertheless, few studies have investigated the relationship between AAPs and the WNT/β-catenin pathway in glaucoma by acting OS and thus, the ROS production. This possible mechanism should be investigated in clinical studies.

Neurotrophins have a major role in cell survival. Several studies have shown that IOP elevation is associated with the inhibition of the retrograde transport of brain-derived-neurotrophic factor (BDNF) which contribute to loss of visual signal [154,155,156]. AAPs, such as risperidone and clozapine, can decrease haloperidol-induced reduction of neurotrophins and can increase BDNF levels [152]. Neurotrophins can control different pathways influencing the activities of GSK3-β and PI3K/Akt pathway [157]. Olanzapine, quetiapine and clozapine, can stimulate in PI3K/Akt pathway and ERK phosphorylation [158]. Recently, clozapine has been shown to directly stimulate ERK phosphorylation in different cell lines through a 5-HT2A receptor-mediated G protein independent pathway [159]. Thus, AAPs could activate neurogenesis. Clozapine can stimulate adult neurogenesis and neuronal survival in hippocampus and PFC regions [160]. Similar to clozapine, other AAPs like quetiapine, olanzapine and aripiprazole have also been shown to increase neural proliferation [161].

Antipsychotics may lead to an added risk of developing POAG, but only in predisposed eyes. Moreover, topiramate has been frequently associated with numerous ocular symptoms, such as acquired myopia and POAG [162]. Unfortunately, drug package inserts are often confusing for clinicians and patients; they simply state “glaucoma” as a contra-indication without further detail [163]. At this time, only a high dose of antipsychotics with high level of anticholinergic and antiadrenergic mechanisms could be a risk factor for angle-closure glaucoma [162]. In comparison, antipsychotics have weaker actions on ocular smooth muscle compared to tricyclic antidepressant and no reports in literacy have shown antipsychotic-induced-angle-closure glaucoma [162]. Muscarinic receptors have been inhibited in glaucoma and lead to the impairment of the visual cortex [164,165]. Nevertheless, many studies have shown that many AAPs may have an antimuscarinic action and could participate in the enhancement of glaucoma. Clozapine and olanzapine present high affinity for the muscarinic receptors by inhibiting it and present an anticholinergic activity [166]. By these mechanisms, AAPs may exacerbate glaucoma process [167]. Thus, the different actions of the AAPs by downregulating both OS and neurotrophins could be unbalanced by their negative role on the muscarinic receptors and may explain that psychotropic medications generally do not affect glaucomatous conditions [162].

## 6. Activation of the Canonical WNT Pathway by Lithium: A Potential Therapeutic Strategy

The dysregulation of GSK-3β is implicated in the pathogenesis of numerous pathologies, such as neuropsychiatric disorders and neurodegenerative diseases [107]. GSK-3β is a controller of numerous pathways including inflammation, neuronal polarity or cell membrane pathways [102]. GSK3β is one of the main inhibitors of the canonical WNT/β-catenin pathway [108]. GSK-3β downregulates the canonical WNT/β-catenin pathway by inhibiting β-catenin cytosolic stabilization and its translocation in the nucleus [168]. Moreover, several studies have shown a link between neuro-inflammation and the augmentation of the GSK-3β pathway and in parallel the diminution of the WNT/β-catenin pathway and the PI3K/Akt pathway [94].

Lithium at concentrations of 1 to 2 mM could downregulate GSK-3β activity [169,170,171]. Lithium diminishes GSK-3β activity through the increase of the inhibitory phosphorylation of GSK3β and by activating the Akt signaling. The stimulation of Akt pathway controls forkhead bow class O (FOXO), Bcl-2 associated death protein (Bad) (a pro-apoptotic protein of the Bcl-2 family) [172,173].

Therapeutic concentrations of the GSK-3β inhibitor lithium involves to the augmentation in β-catenin levels [174,175] and then leads to β-catenin transcriptional activity [11,176]. In mouse brains, the activation of β-catenin levels could have anti-depressant-like actions of lithium [177] whereas the inhibition of β-catenin implicate a depression-like phenotype [178,179].

## 7. Lithium and the Different Altered Pathways Involved in Glaucoma

**Figure 2:**
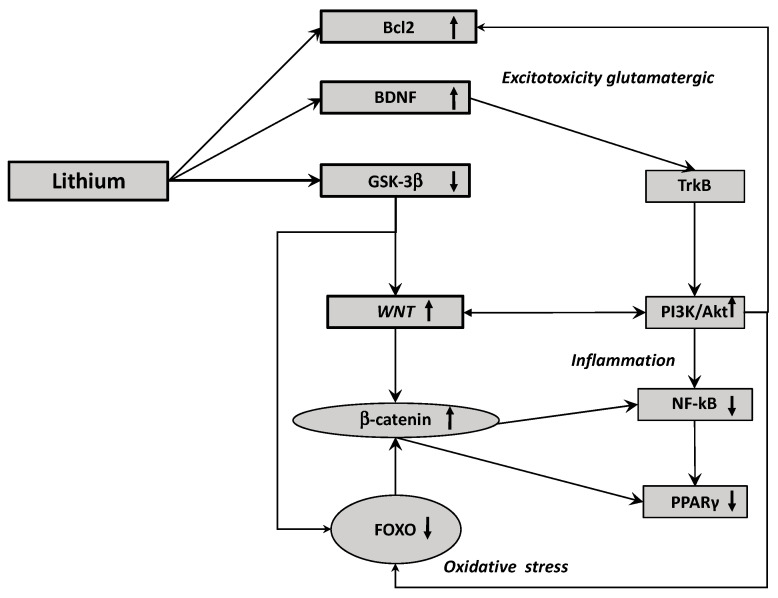
Underlying molecular mechanisms of lithium actions in glaucoma. BDNF: brain-derived neurotrophic factor; FOXO: forkhead box class O; GSK-3β: glycogen synthase kinase-3β; LRP 5/6: low-density lipoprotein receptor-related protein 5/6; NF-ϰB: nuclear factor-kappa B; PI3K-Akt: phosphatidylinositol 3-kinase-protein kinase B; TrkB: tropomyosin receptor kinase B.

No studies have directly focused on the interest of lithium in glaucoma by targeting the OS. However, the energy metabolisms implicated in OS are mainly controlled by the intracellular FOXO transcription factors (FOXO1, 3a, 4) [180]. The interaction between β-catenin and FOXO transcription factors can lead to cell quiescence and cell cycle stop. Β-catenin inhibits its transcriptional complex with TCF/LEF by interacting with FOXO-induced ROS [181]. β-catenin does not translocate to the nucleus and accumulates in the cytoplasm to inactivate the WNT/β-catenin pathway [182,183]. Some studies have shown that lithium can diminish FOXO3a transcriptional activity and can diminish the level of active FOXO3a [184]. Thus, through the downregulation of GSK3-β pathway, stimulating the WNT/β-catenin pathway and diminishing the FOXO, lithium may participate to the reduction of OS (Figure 2). 

Lithium can activate both Bcl2 and BNDF to diminish the excitotoxicity glutamate pathway. By inactivating GSK3-β, lithium can upregulate the WNT pathway leading to the diminution of FOXO and then, to reduce the oxidative stress. By activating the WNT pathway and then the PI3K/Akt pathway, lithium can reduce inflammation by decreasing the expression of NF-kB pathway and PPARγ.

Moreover, numerous in vitro studies have presented that lithium administration may downregulate hydrogen peroxide-induced cell death as well as obstruct lipid peroxidation and protein oxidation in cortical cells [185,186,187,188,189,190]. Furthermore, lithium can act as an anti-oxidant by increasing CHS levels in neurons of rat dopaminergic N27 [186,190].

### 7.1. Lithium and Inflammation

By inhibiting the GSK-3β activity and thus increasing the WNT/β-catenin pathway, the lithium administration could implicate a decrease of the neuro-inflammation by controlling the NF-ϰB pathway. The stimulation of the WNT pathway cascade diminishes inflammation and involves the neuroprotection through interactions between microglia/macrophages and astrocytes [191,192].

Numerous findings have presented a negative interplay between WNT/β-catenin pathway and NF-ϰB pathway [193]. The NF-ϰB transcription factor family belongs of five members in the cytosol under non-activated conditions: NF-ϰB1 (p50/p105), NF-ϰB 2 (p52/p100), RelA (p65), RelB and c-Rel [194]. Β-catenin can complex with RelA and p50 to diminish the activity of the NF-ϰB pathway [195]. Furthermore, through the interaction with the PI3K, β-catenin can diminish the activity of NF-ϰB pathway [196]. This inhibitory role of β-catenin on NF-ϰB pathway activity was shown in several cell types, including fibroblasts, epithelial cells, hepatocytes and osteoblasts [193]. Moreover, the stimulation of GSK-3β inhibits the β-catenin and activates the NF-ϰB pathway [197]. The potential protective role of β-catenin was due to the stimulation of PI3K/Akt pathway and thus the diminution of TLR4-driven inflammatory response in hepatocytes [198]. NF-ϰB pathway stimulation inhibits the complex β-catenin/TCF/LEF by the activation of LZTS2 in cancer cells [199]. DKK1, a WNT inhibitor, was a target gene of the NF-ϰB pathway leading to a negative interplay to decrease the β-catenin signaling [200] (Figure 2).

A recent study has shown that the WNT pathway was one of the major process of action of lithium in adipose cells, and this interaction is done by the diminution of PPARγ expression [201]. PPARs are ligand-activated transcription factors which bind PPRE (PPAR-response elements). PPARs are involved in several disease processes, including cell differentiation, proteins metabolism, lipids metabolism, carcinogenesis [202,203], adipocyte differentiation, insulin sensitivity, and inflammation [204,205]. PPARγ ligands, such as thiazolidinediones (TZDs), are able to diminish inflammation process [206].

A negative interplay was well described between PPARγ and the WNT pathway [108,207,208,209]. The PI3K/Akt pathway, which is activated by β-catenin [112,210], interacts by phosphorylating GSK-3β to negatively control the expression of PPARγ [211]. PPARγ agonists diminish β-catenin expression through the activation of GSK-3β [212]. Moreover, PPARγ agonists stimulate Dickkopf-1 (DKK1) to diminish the canonical WNT/β-catenin pathway and then to downregulate fibroblasts differentiation [213]. Furthermore, PPARγ agonists activate GSK-3β to decrease β-catenin expression [212].

### 7.2. Lithium and Glutamatergic Pathway

Lithium has been also associated with an influence in levels of pro-apoptotic proteins. Bax, named Bcl-2 associated C protein, is a key modulator promoting apoptosis by binding to and antagonizing the Bcl-2 protein. The tumor suppressor protein, p53, targets Bcl-2 and Bax and then promotes growth arrests and cell death in response to cell damage [214].

Several studies have demonstrated that the neuroprotective effects of lithium could be attributed to increased Bcl-2 levels. Indeed, lithium therapy of cultured cerebellar granule cells increased mRNA and protein levels of Bcl-2, the Bcl-2/Bax protein level ration increased by 5-fold after treatment duration for 5 to 7 days [122]. The stimulation in Bcl-2 expression involves neurogenesis in the hippocampus and entorhinal cortex in mice by the stimulation of axon diameters and neurite growth on the CA3 area of the hippocampus and increase myelination in the entorhinal cortex [215]. Lithium can stimulate anti-apoptotic-increasing Bcl-2 levels and can reduce Bax activity [216]. The phosphorylation of Bcl 2 at serine 70 is required for a complete anti-apoptotic action [217] and lithium have this ability [218]. Lithium inhibits Bcl-2 dephosphorylation and caspase-2 activation through the reduction of the protein phosphatase-2A activity [218] (Figure 2).

Glutamate excitotoxicity has been associated with the upregulation of Bax and p53 and the diminution of Bcl-2 [122]. The apoptosis role of the glutamate was associated with the stimulation of activator protein-1 (AP-1) stimulated by the activation of c-Jun N-terminal kinase (JNK) and p38 mitogen-activated protein kinase (MAP kinase) and phosphorylation of c-Jun and p53 [219].

Through the diminution of the GSK-3β activity, lithium activates as a powerful controller of both EAAT3 and NMDA receptors [220]. Furthermore, a direct possible way may be the diminution of presynaptic NMDA receptors and then the stimulation of postsynaptic AMPA receptors by glutamate release. This process is followed by the stimulation of the influx of calcium and secretion of brain-derived neurotrophic factor (BDNF). Lithium can activate the release of the excitatory neurotransmitter and glutamate, from cerebral cortex slices [221]. This release was associated by the stimulation of inositol 1,4,5-trisphosphate [Ins(1,4,5)P_3_] accumulation. The stimulation in Ins(1,4,5)P_3_ accumulation was involved by the selective stimulation of the *N*-methyl-d-aspartate (NMDA) receptor/channel by glutamate. The upregulation of the NMDA receptor is known to lead in increase Ins(1,4,5)P_3_ accumulation [222]. Then, BDNF activates the receptor tyrosine kinase B (TrkB) leading to neuronal survival and differentiation [223].

The stimulation of BDNF-TrkB pathway activates the Akt/mTOR pathway leading to the activation of the WNT/β-catenin pathway and to the enhancement of synaptic proteins [224]. Few therapeutic levels of lithium stimulate the BDNF-TrkB pathway and then the Akt/mTOR pathway to protect neurons from glutamate excitotoxicity [225]. Lithium downregulates excessive glutamate, NMDA receptor-mediated calcium influx in neurons, and diminishes NR2B subunit tyrosine phosphorylation by the Src/Fyn kinase [226].

PPARγ antagonists can stop the stimulation of PPARγ DNA binding activity and antioxidant enzymatic activities (SOD) downregulating the protection of PPARγ activation in OGD-exposed neurons [227]. Other processes by which these PPARγ agonists can prevent OS include a diminution in iNOS activity, NFκB blockade, inhibition of TNF-α release, or activation of nuclear factor (erythroid-derived 2)-like 2 (Nrf2) [228]. By the negative crosstalk between WNT and PPARγ, Lithium administration by inhibiting the GSK-3β could act as a PPARγ antagonist and leads to increase the WNT pathway resulting in the diminution of oxidative stress. 

## 8. Conclusions

Currently, few investigations have studied lithium as a possible alternative therapeutic way to treat glaucoma patients. Nevertheless, lithium, in low doses, appears to be helpful for treating glaucoma by targeting oxidative stress, inflammation, and the glutamatergic pathway. The action of lithium is mainly involved by its negative interaction with GSK-3β, the main inhibitor of the WNT/β-catenin pathway. In glaucoma, the WNT/β-catenin is downregulated to allow the stimulation of oxidative stress, inflammation, and glutamatergic pathway. Stimulating the WNT/β pathway, through the inhibition of GSK-3β, lithium, could be an innovative therapeutic way in glaucoma. In current clinical practice, lithium is coupled with AAPs. AAPs have a hypotension effect but little impact on the glaucoma process. Future prospective studies should focus on lithium and its different actions in glaucoma and the possible effects of the association lithium-AAPs in this disease. 

## Data Availability

Not applicable.
